# Coupling Models of Resistive Valves to Muscle Mechanics in Cardiac Fluid–Structure Interaction Simulations

**DOI:** 10.1002/cnm.70119

**Published:** 2025-12-04

**Authors:** Michele Bucelli, Luca Dede

**Affiliations:** ^1^ Dipartimento di Matematica, MOX Laboratory of Modeling and Scientific Computing Milano Italy

**Keywords:** cardiac modeling, fluid–structure interaction, resistive immersed implicit surface, valve modeling

## Abstract

To accurately simulate all phases of the cardiac cycle, computational models of hemodynamics in heart chambers need to include a sufficiently faithful model of cardiac valves. This can be achieved efficiently through resistive methods, and the resistive immersed implicit surface (RIIS) model in particular. However, the conventional RIIS model is not suited to fluid–structure interaction (FSI) simulations, since it neglects the reaction forces by which valves are attached to the cardiac walls, leading to models that are not consistent with Newton's laws. In this paper, we propose an improvement to RIIS to overcome this limitation, by adding distributed forces acting on the structure to model the attachment of valves to the cardiac walls. The modification has a minimal computational overhead thanks to an explicit numerical discretization scheme. Numerical experiments in both idealized and realistic settings demonstrate the effectiveness of the proposed modification in ensuring the physical consistency of the model, thus allowing us to apply RIIS and other resistive valve models in the context of FSI simulations.

## Graphical abstract

Scheme of the cardiac fluid‐structure interaction model including valve‐wall interaction.

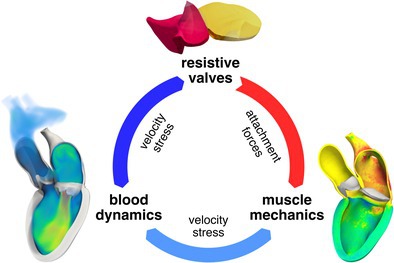

## 1 Introduction

The motion of cardiac valves, both native and prosthetic, is regulated by the complex and dynamic interplay of blood flow with the mechanics of valve leaflets. A physically detailed computational model of valves must be able to deal with large deformations, the effect of chordae tendineae, and the contact among valve leaflets themselves [1, 2, 3, 4, 5, 6, 7, 8, 9, 10, 11]. These aspects, although crucial when focusing on valve dynamics or valvular dysfunctions, pose a significant challenge in terms of model calibration, complexity, and computational cost.

As an alternative, it is possible to use valve models that are simplified, geometrically (e.g., using a planar representation of the valve), physically (by neglecting fluid–structure interaction [FSI]), or both [12, 13, 14]. This is especially convenient for models that do not focus on the valves themselves, but only need to account for the macroscopic effect of the valves on the flow within heart chambers, such as the way their opening and closing determines heartbeat phases [15], or the large‐scale jets and vortices caused by valve leaflets [16, 17]. Simplified models may be sufficiently accurate to capture these features, yet at a manageable complexity and cost.

Many such models are based on a resistive approach, by which the equations for fluid flow are modified to penalize the mismatch between the fluid velocity and valve velocity in the proximity of the valve. This corresponds to modeling the valve as a porous medium with very low permeability [18]. For example, valves are modeled in Reference [19] through Navier–Stokes–Brinkman (NSB) equations, assuming a planar geometry. The resistive immersed surface (RIS) method [20, 21, 22] represents valves as resistive surfaces, which are assumed to be conforming to the mesh used to discretize fluid equations. In both NSB and RIS, the opening and closing of the valve may be modeled by varying the associated penalization coefficient (i.e., its permeability) over time.

In this work, we consider the resistive immersed implicit surface (RIIS) method [23], which relaxes the mesh‐conformity constraint of RIS by implicitly representing valves through signed distance functions. This allows us to represent the valve leaflets as they open and close, regardless of the underlying mesh. The RIIS method was extensively applied to cardiovascular hemodynamics simulations [15, 16, 24, 25, 26, 27, 28, 29, 30], showcasing its effectiveness in supporting detailed and patient‐specific [31, 32, 33, 34, 35] computational models.

However, in the specific context of models that include FSI between blood and the cardiac walls, the RIIS method (and resistive methods in general) may lead to issues in the balance of forces. Indeed, resistive surfaces exert a force on the fluid, to enforce the no‐slip condition in proximity to the valve. In real hearts, this force is sustained by the attachment of valves to the walls, through either the chordae tendineae or the valve annuli. By simply applying the RIIS method in its original form, this is neglected, which leads to a force imbalance and possibly to an unphysical behavior of the FSI system as a whole. This can be compensated through an ad‐hoc calibration of other parameters, most notably the boundary conditions on the mechanics model, as done in Reference [15]. However, this approach lacks physical soundness: the application of standard RIIS in the FSI context leads to models that do not satisfy Newton's laws.

In this paper, we propose an improvement to the RIIS method for FSI that accounts for the forces keeping the valves attached to the cardiac walls. After deriving an expression for these forces, we demonstrate through numerical experiments how neglecting them may lead to unphysical displacements, and how the proposed modification eliminates this issue. We remark that, with minor modifications, the proposed method can be extended to other resistive valve models, which may face the same issue when combined with FSI simulations.

The remainder of this paper is structured as follows. In Section 2, we present the mathematical models used for cardiac FSI, introduce the modifications for coupling resistive valves to solid mechanics, and discuss numerical discretization. Section 3 illustrates the properties of the proposed modification through numerical experiments. Finally, Section 4 draws some conclusive remarks.

## 2 Models and Methods

This section describes the modeling and discretization framework employed for cardiac FSI simulations, and presents the approach proposed in this work to couple resistive valves and muscular mechanics.

### 2.1 Cardiac Fluid–Structure Interaction Modeling

Let $t\in \left(0,T\right)$ denote the independent time variable, with T>0 denoting the final time. Let $\Omega \subset {\mathbb{R}}^{3}$ be an open set, moving over time, representing the volume occupied by a beating heart. We partition Ω into two subsets, $\Omega_{f}$ and $\Omega_{s}$, representing the volume occupied by the blood inside the heart cavities and by the cardiac walls, respectively. Notice that the set $\Omega_{s}$ does not include the cardiac valve leaflets, which are instead accounted for through a resistive approach, and are thus immersed in $\Omega_{f}$, as detailed below. Let $\Sigma =\partial \Omega_{f}\cap \partial \Omega_{s}$ denote the fluid–solid interface.

To track the deformation of the domain in time, we introduce the fixed reference domain $\hat{\Omega }\subset {\mathbb{R}}^{3}$, as well as its subsets ${\hat{\Omega }}_{f}$ and ${\hat{\Omega }}_{s}$. The current domains $\Omega_{f}$ and $\Omega_{s}$ are related to the reference domains through the continuous maps [15]

$$\begin{array}{l} \psi_{f}:{\hat{\Omega }}_{f}\times \left(0,T\right)\to \Omega_{f},\\ \psi_{s}:{\hat{\Omega }}_{s}\times \left(0,T\right)\to \Omega_{s}. \end{array}$$

Henceforth, we shall use a hat to denote functions and sets in the reference configuration, without a hat their counterparts mapped onto the current configuration, and vice versa. Similarly, $\hat{x}$ denotes points in the reference domain $\hat{\Omega }$, and x denotes points in the current domain Ω.

We consider an FSI model of the displacement of the cardiac walls and the blood flow [15, 36], based on the arbitrary Lagrangian–Eulerian (ALE) formulation of incompressible Navier–Stokes equations [37, 38] and on a hyperelastic model for muscular mechanics, including active stress [39]. The model features the following unknowns:

$$\begin{array}{rr} {\hat{d}}_{\mathrm{ALE}}:{\hat{\Omega }}_{f}\times \left(0,T\right)\to {\mathbb{R}}^{3} & \text{fluid domain displacement},\\ u:\Omega_{f}\times \left(0,T\right)\to {\mathbb{R}}^{3} & \text{blood velocity},\\ p:\Omega_{f}\times \left(0,T\right)\to \mathbb{R} & \text{blood pressure},\\ \hat{d}:{\hat{\Omega }}_{s}\times \left(0,T\right)\to {\mathbb{R}}^{3} & \text{solid displacement}. \end{array}$$

The fluid domain displacement is computed by extending the solid displacement from the fluid–solid interface $\hat{\Sigma }$ to the whole ${\hat{\Omega }}_{f}$, by solving the following problem [40]:

$$\begin{array}{c} \left\{\begin{array}{cc} {ℒ}_{\mathrm{ALE}}\left({\hat{d}}_{\mathrm{ALE}}\right)=0 & \text{in}\;{\hat{\Omega }}_{f}\times \left(0,T\right),\\ \;{\hat{d}}_{\mathrm{ALE}}=\hat{d} & \mathrm{on}\;\hat{\Sigma }\times \left(0,T\right),\\ \;{\hat{d}}_{\mathrm{ALE}}=0 & \mathrm{on}\;\left(\partial {\hat{\Omega }}_{f}\backslash \hat{\Sigma }\right)\times \left(0,T\right), \end{array}\right. \end{array}$$

where ${ℒ}_{\mathrm{ALE}}$ is a suitable differential operator. At any time $t\in \left(0,T\right)$, the fluid domain in its current configuration is obtained by setting

$$\psi_{f}\left(\hat{x},t\right)=\hat{x}+{\hat{d}}_{\mathrm{ALE}}\left(\hat{x},t\right),$$

$$\Omega_{f}=\left\{x\in {\mathbb{R}}^{3}:x=\psi_{f}\left(\hat{x},t\right),\;\hat{x}\in {\hat{\Omega }}_{f}\right\}.$$

The evolution of blood velocity and pressure is prescribed by the incompressible Navier–Stokes equations, in ALE formulation:

(1) $$\begin{array}{c} \left\{\begin{array}{cc} \rho_{f}\frac{\partial u}{\partial t}+\rho_{f}\left(\left(u-u_{\mathrm{ALE}}\right)\cdot \text{∇}\right)u-\text{∇}\cdot \sigma_{f}\left(u,p\right)+R\left(u,u_{\mathrm{ALE}}\right)=0 & \text{in}\;\Omega_{f}\times \left(0,T\right)\;,\\ \;\text{∇}\cdot u=0 & \text{in}\;\Omega_{f}\times \left(0,T\right). \end{array}\right. \end{array}$$

In the above, $\rho_{f}$ is the blood density, $\sigma_{f}\left(u,p\right)=\mu \left(\text{∇}u+{\text{∇}}^{⊤}u\right)-\mathrm{pI}$ is the Cauchy stress tensor, with μ the blood viscosity, and $u_{\mathrm{ALE}}:\Omega_{f}\times \left(0,T\right)\to {\mathbb{R}}^{3}$ is the known domain velocity, defined as

$$u_{\mathrm{ALE}}\left(x,t\right)=\frac{\partial {\hat{d}}_{\mathrm{ALE}}}{\partial t}.$$

The term $R\left(u,u_{\mathrm{ALE}}\right)$ accounts for the presence of cardiac valves through the RIIS method [23, 24, 31].

Let $N_{v}$ be the number of valves present in the model. Each valve is represented in reference configuration by a surface ${\hat{\Gamma }}_{k}$ (for $k=1,\ldots ,N_{v}$) immersed in the fluid domain (i.e., such that $\Gamma_{k}\cap {\hat{\Omega }}_{f}\neq \emptyset$). Each surface ${\hat{\Gamma }}_{k}$ moves following the fluid domain displacement ${\hat{d}}_{\mathrm{ALE}}$, and it opens and closes according to a prescribed displacement field $d_{k}:{\hat{\Gamma }}_{k}\times \left(0,T\right)\to {\mathbb{R}}^{3}$. Therefore, at any time $t\in \left(0,T\right)$, the current configuration of the valve is given by

$$\Gamma_{k}=\left\{x\in {\mathbb{R}}^{3}:x=\hat{x}+{\hat{d}}_{\mathrm{ALE}}\left(\hat{x},t\right)+d_{k}(\hat{x},t),\hat{x}\in {\hat{\Gamma }}_{k}\right\}.$$

The resistive term $R\left(u,u_{\mathrm{ALE}}\right)\in {\mathbb{R}}^{3}$ in (1) has the purpose of weakly enforcing a no‐slip condition in the vicinity of the valve surface. Following References [15, 24], we neglect the valve velocity due to changes in valve configuration, which corresponds to assuming $\frac{\partial d_{k}}{\partial t}\approx 0$. Therefore, the valve velocity equals $u_{\mathrm{ALE}}$, and the resistive term must penalize the mismatch between u and $u_{\mathrm{ALE}}$. It is defined as:

(2) $$R\left(u,u_{\mathrm{ALE}}\right)=\sum_{k=1}^{N_{v}}R_{k}\left(u,u_{\mathrm{ALE}}\right),$$

(3) $$R_{k}\left(u,u_{\mathrm{ALE}}\right)=\frac{R_{k}}{\varepsilon_{k}}\delta_{k}\left(\varphi_{k}\left(x\right)\right)\left(u-u_{\mathrm{ALE}}\right).$$

In the above, $R_{k}>0$ is the valve resistance, $\varepsilon_{k}>0$ is the half‐thickness of the k‐th valve, $\varphi_{k}:\Omega \to \mathbb{R}$ is the signed distance function from the current configuration of the surface $\Gamma_{k}$, and $\delta_{k}$ is a smoothed Dirac delta function, defined as

$$\begin{array}{c} \delta_{k}\left(y\right)=\left\{\begin{array}{cc} \frac{1}{2\varepsilon_{k}}\left(1+\cos\left(\frac{\mathrm{\pi y}}{\varepsilon_{k}}\right)\right) & \text{if}|y|\;\leq \varepsilon ,\\ \;0 & \text{otherwise}. \end{array}\right. \end{array}$$

The evolution of muscular displacement is prescribed by the following elastodynamics equation in Lagrangian reference:

(4) $$\rho_{s}\frac{\partial^{2}\hat{d}}{\partial t}-\text{∇}\cdot P_{s}\left(\hat{d},t\right)=0\;\text{in}\;{\hat{\Omega }}_{s}\times \left(0,T\right),$$

wherein $P_{s}$ is the first Piola‐Kirchhoff stress tensor. The stress tensor is decomposed as the sum of an active and a passive contribution, that is.

$$P_{s}\left(\hat{d},t\right)=P_{s}^{\mathrm{act}}\left(\hat{d},t\right)+P_{s}^{\mathrm{pas}}\left(\hat{d}\right).$$

The passive stress $P_{s}^{\mathrm{pas}}$ accounts for the elastic response through a hyperelastic constitutive law [39, 41, 42], while the active stress $P_{s}^{\mathrm{act}}$ takes into account the contractile force [43, 44]. The displacement field $\hat{d}$ defines the current configuration of the solid subdomain through

$$\begin{array}{c} \psi_{s}\left(\hat{x},t\right)=\hat{x}+\hat{d}\left(\hat{x},t\right)\;,\\ \Omega_{s}=\left\{x\in {\mathbb{R}}^{3}:x=\psi_{s}\left(\hat{x},t\right),\;\hat{x}\in {\hat{\Omega }}_{s}\right\}. \end{array}$$

Finally, fluid and solid are coupled at their interface Σ by prescribing the continuity of velocity and stress, that is [36, 45]

$$\left\{\begin{array}{cc} u=\frac{\partial d}{\partial t} & \mathrm{on}\;\Sigma ,\\ \sigma_{f}\left(u,p\right)n=\frac{1}{J}{\mathrm{FP}}_{s}{\left(d,t\right)}^{⊤}n & \mathrm{on}\;\Sigma , \end{array}\;\right.$$

where $\hat{F}=I+\hat{d}$, $\hat{J}=\det\hat{F}$, F and J are their counterparts in the deformed configuration, and n is the unit vector normal to Σ, outgoing from $\Omega_{f}$.

To close the problem, both the fluid and solid equations are endowed with suitable boundary and initial conditions [15, 24, 42].

### 2.2 RIIS‐Mechanics Coupling

The resistive terms $R_{k}$ in (3) can be interpreted as force densities exerted by the fluid on the valves. Indeed, these forces provide a simplified representation of the interface forces exchanged by blood and valve leaflets. In real hearts, the leaflets are attached to the cardiac walls, to which these forces are transmitted. The model discussed in Section 2.1 neglects this attachment, thus leading to an imbalance of forces in the FSI system. In other words, the system as a whole does not satisfy Newton's laws.

**FIGURE 1:**
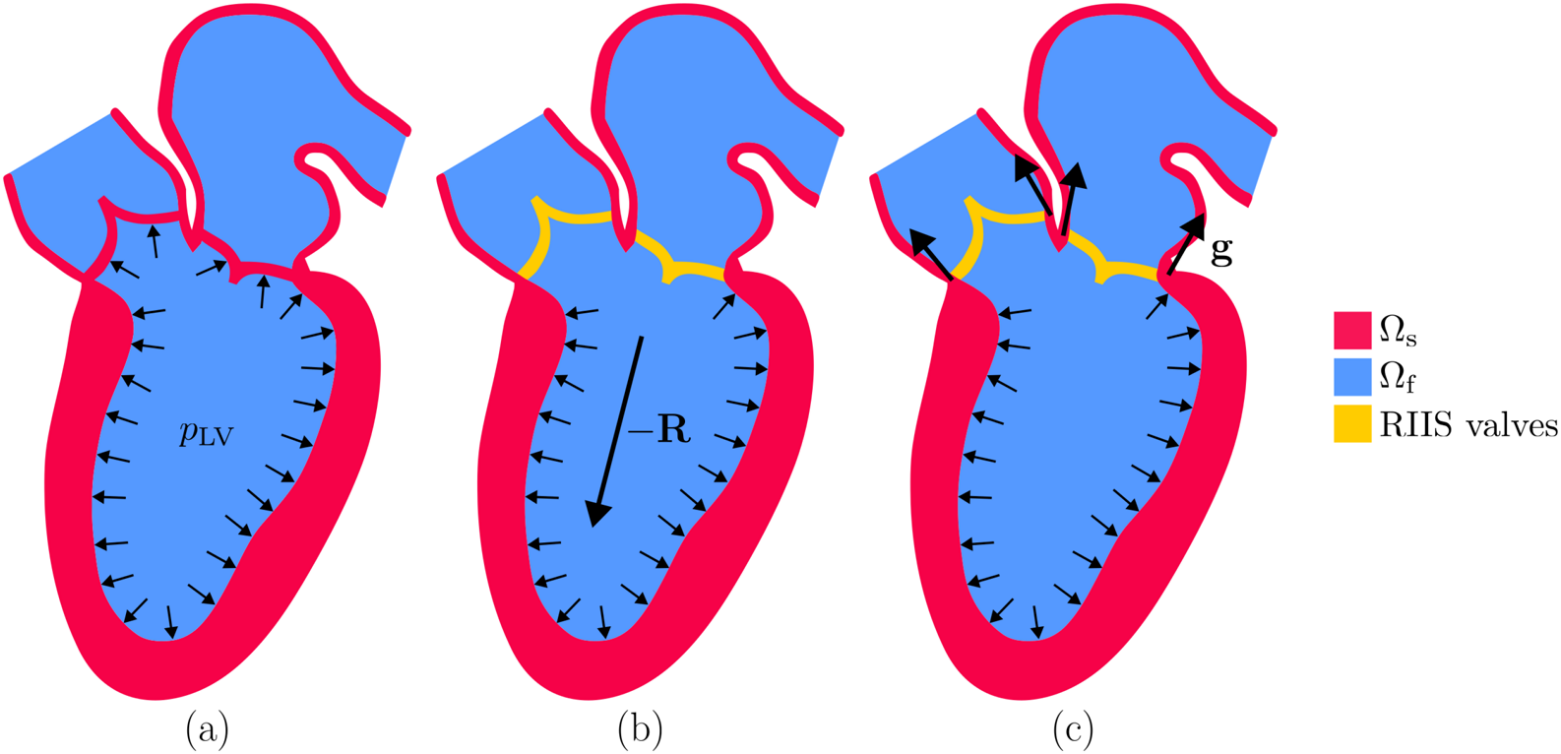
(a) When valves are attached to the wall, the resultant of pressure forces is zero (assuming that the pressure is constant within the chamber). (b) If the valves are represented through RIIS, the forces they are subject to do not transfer to the wall, and thus the forces acting on the wall have a non‐zero resultant. (c) Including the forces g to account for the attachment of valves to the wall, the equilibrium is restored.

This can lead to unphysical results. Consider, for example, a ventricular chamber during an isovolumetric phase, when both valves are closed. If we assume that the pressure is uniform within the chamber, then the pressure forces that act on the endocardium and on the valves have a null resultant, and thus do not lead to a net acceleration of the chamber walls (Figure 1a). This is consistent with the fact that the pressure forces are internal to the FSI system. However, if we apply the model discussed in Section 2.1, the forces exerted by the fluid on the valve leaflets do not act on the solid, and the remaining pressure forces acting on the chamber walls now have a non‐zero resultant (Figure 1b). This may lead to an acceleration of the FSI system, even though there are no external forces acting on it. This imbalance is also present if the pressure is not homogeneous within the chamber. We demonstrate this behavior in Section 3.

To address this issue, we modify the solid mechanics Equation (4) by adding a distributed force $\hat{g}:{\hat{\Omega }}_{s}\times \left(0,T\right)\to {\mathbb{R}}^{3}$, accounting for the attachment forces between the valves and the walls:

$$\rho_{s}\frac{\partial \hat{d}}{\partial t}-\text{∇}\cdot P_{s}\left(\hat{d},t\right)=\hat{J}\hat{g}.$$

We assume that the force $\hat{g}$ can be expressed as the sum of one force for each valve, denoted by ${\hat{g}}_{k}:{\hat{\Omega }}_{s}\times \left(0,T\right)\to {\mathbb{R}}^{3}$ for $k=1,\ldots ,N_{v}$:

$$\hat{g}\left(\hat{x},t\right)=\sum_{k=1}^{N_{v}}{\hat{g}}_{k}\left(\hat{x},t\right).$$

To derive an expression for the forces ${\hat{g}}_{k}$, we make the following assumptions.

Assumption 1 In the deformed configuration, each surface $\Gamma_{k}$ is in contact with the structure domain, that is $\Gamma_{k}\cap \Omega_{s}\neq \emptyset$.

Assumption 2 Each valve remains in equilibrium at all times, meaning the net force acting on it is zero.

Assumption 3 The spatial distribution of the force ${\hat{g}}_{k}$ is the same as in the fluid, that is there exists a function $C_{k}:\left(0,T\right)\to {\mathbb{R}}^{3}$ such that

$${\hat{g}}_{k}=C_{k}\left(t\right)\delta_{k}\left(\varphi_{k}\left(\hat{x}+\hat{d}\right)\right).$$

Notice that this is different from zero owing to Assumption 1.

By Assumption 2, we choose $C_{k}\left(t\right)$ such that, at every time t, the resultant of ${\hat{g}}_{k}$ equals the force that the fluid exerts on the k‐th valve, denoted by $F_{k}$:

$$F_{k}\left(t\right)=\int_{\Omega_{s}}g_{k}\;dx=\int_{{\hat{\Omega }}_{s}}\hat{J}{\hat{g}}_{k}\;d\hat{x}=C_{k}\left(t\right)\int_{{\hat{\Omega }}_{s}}\hat{J}\delta_{k}\left(\varphi_{k}\left(\hat{x}+\hat{d}\right)\right)\;d\hat{x}.$$

Introducing

$$V_{k}\left(t\right)=\int_{{\hat{\Omega }}_{s}}\hat{J}\delta_{k}\left(\varphi_{k}\left(\hat{x}+\hat{d}\right)\right)\;d\hat{x},$$

we obtain:

$$\begin{array}{c} C_{k}\left(t\right)=\frac{F_{k}\left(t\right)}{V_{k}\left(t\right)},\\ {\hat{g}}_{k}\left(\hat{x},t\right)=\frac{F_{k}\left(t\right)}{V_{k}\left(t\right)}\delta_{k}\left(\varphi_{k}\left(\hat{x}+\hat{d}\right)\right). \end{array}$$

Notice that $V_{k}\left(t\right)>0$ for all t>0 by Assumption 1.

The forces $F_{k}\left(t\right)$ are readily computed by integrating the resistive term of Navier–Stokes momentum equation:

$$F_{k}\left(t\right)=\int_{\Omega_{f}}R_{k}\left(u,u_{\mathrm{ALE}}\right)\;dx.$$

With a slight abuse of notation, we will henceforth denote the forces acting on the solid with $\hat{g}\left(d,u,t\right)$ and ${\hat{g}}_{k}\left(d,u,t\right)$, to emphasize their dependence on the solid displacement (and its derivatives) and on the fluid velocity.

As a final remark, we point out that Assumption 2 amounts to neglecting the valves' inertia. This assumption can be relaxed by modifying the definition of $F_{k}$ to

$$F_{k}\left(t\right)=\int_{\Omega_{f}}R\left(u,u_{\mathrm{ALE}}\right)dx-\int_{\Omega_{f}}\delta \left(\varphi_{k}\left(x\right)\right)\rho_{v}\frac{\partial u_{\mathrm{ALE}}}{\partial t}dx,$$

where $\rho_{v}$ is the surface density of valves. In our numerical experiments, we found that the second integral in the above equation, that is, the inertial contribution, is negligible with respect to the first term, that is, the resistive contribution. Accordingly, we chose to neglect it in the interest of model simplicity. Nonetheless, the inertial term might play a more prominent role in scenarios involving rapid valve movement, which are not considered here.

### 2.3 Valve‐Wall Coupling in Electromechanical Models

It is worth observing that issues in the balance of the pressure forces acting in chamber walls may also arise in models of cardiac electromechanics, that is models in which hemodynamics is surrogated with lumped‐parameters circulation models. For ventricular models where the domain is artificially cut at the base of the ventricle, this can be addressed by the so‐called energy‐consistent boundary conditions [39, 46], which make sure that the boundary forces exerted on the ventricular base balance the pressure forces acting on the walls. Alternatively, the force balance can be ensured by adding solid discs in place of the valves [42, 47, 48, 49, 50]. These, however, are not practical for models involving three‐dimensional fluid dynamics. The model proposed in this work can be seen as an extension of these techniques to FSI models.

### 2.4 Discretization and Solvers

We introduce a partition of the time domain $\left(0,T\right)$ into intervals $\left(t^{n},t^{n+1}\right)$, where $t^{n}=n\Delta t$, with Δt>0 denoting the time discretization step. We denote with a superscript n the time‐discrete approximation of a function at time $t^{n}$, for example, $u^{n}\approx u\left(t^{n}\right)$. We approximate time derivatives through first‐order backward finite difference formulas for both the fluid and the solid model. To address the coupling of fluid displacement, fluid and solid, we consider the geometrically‐explicit monolithic scheme discussed in Reference [36], suitably adapted to account for resistive modeling of valves.

The newly added term $\hat{g}$ is nonlinear with respect to the solid displacement d, and it introduces a further source of fluid–solid coupling in the problem, through the dependency of the force density $\hat{g}$ on the fluid velocity u in the vicinity of the immersed surfaces. For its numerical discretization, we use an explicit treatment, that is we compute the valve forces using the fluid velocity, pressure and solid displacement from previous time step. This facilitates the convergence of the nonlinear solver, without causing instability in our numerical experiments (see Section 3). We remark that this choice introduces an error of the first order with respect to the time step size Δt, which might hinder the accuracy of higher order time discretization schemes. This limitation can be overcome by using a suitable higher‐order extrapolation in computing $\hat{g}$.

The resulting time discrete problem reads:

$$\begin{array}{c} \left\{\begin{array}{c} \begin{array}{cc} L_{\mathrm{ALE}}\left({\hat{d}}_{\mathrm{ALE}}^{n+1}\right)=0 & \text{in}\;{\hat{\Omega }}_{f},\\ \;{\hat{d}}_{\mathrm{ALE}}^{n+1}={\hat{d}}^{n} & \mathrm{on}\;\hat{\Sigma },\\ \;{\hat{d}}_{\mathrm{ALE}}^{n+1}=0 & \mathrm{on}\;\partial {\hat{\Omega }}_{f}\backslash \hat{\Sigma },\\ \;{\hat{u}}_{\mathrm{ALE}}^{n+1}=\frac{{\hat{d}}_{\mathrm{ALE}}^{n+1}-{\hat{d}}_{\mathrm{ALE}}^{n}}{\Delta t} & \text{in}\;{\hat{\Omega }}_{f}\\ \;\begin{array}{c} \rho_{f}\frac{u^{n+1}-u^{n}}{\Delta t}+\rho_{f}\left(\left(u^{n}-u_{\mathrm{ALE}}^{n+1}\right)\cdot \nabla \right)u^{n+1}\\ \;-\nabla \cdot \sigma_{f}\left(u^{n+1},p^{n+1}\right)+R\left(u^{n+1},u_{\mathrm{ALE}}^{n+1}\right)=0 \end{array} & \text{in}\;\Omega_{f}^{n+1},\\ \;\nabla \cdot u^{n+1}=0 & \text{in}\;\Omega_{f}^{n+1},\\ \rho_{s}\frac{{\hat{d}}^{n+1}-2{\hat{d}}^{n}+{\hat{d}}^{n-1}}{\Delta t^{2}}-\nabla \cdot P_{s}\left({\hat{d}}^{n+1},{\hat{s}}^{n+1}\right)={\hat{J}}^{n}\hat{g}\left({\hat{d}}^{n},u^{n},t^{n}\right) & \text{in}\;{\hat{\Omega }}_{s},\\ \;u^{n+1}=\frac{d^{n+1}-d^{n}}{\Delta t} & \mathrm{on}\;\Sigma^{n+1},\\ \sigma_{f}\left(u^{n+1},p^{n+1}\right)n^{n+1}=\frac{1}{J^{n+1}}F^{n+1}P_{s}{\left(d^{n+1},s^{n+1}\right)}^{⊤}n^{n+1} & \mathrm{on}\;\Sigma^{n+1}, \end{array} \end{array}\right. \end{array}$$

endowed with appropriate boundary and initial conditions for both fluid and solid. This time discretization scheme is first‐order accurate with respect to Δt, due to explicit geometric coupling [36], the finite difference approximation of time derivatives, and the explicit computation of the force g.

Finally, the problem is discretized in space using finite elements, with piecewise linear polynomials on a tetrahedral or hexahedral mesh, for all the problems involved. Navier–Stokes equations are stabilized using the VMS‐LES method [51], modified to account for the presence of the resistive term R [15, 24]. We linearize the coupled fluid–solid system through Newton's method and then solve it with the preconditioned GMRES method [36]. The models and methods described in this paper were implemented using life ^x^ ^1^ [52, 53, 54], a C++ library for the simulation of multiphysics problems for cardiac applications, based on deal.II ^2^ [55, 56].

## 3 Numerical Results

This section presents numerical experiments that demonstrate the effect of the proposed model modification. Table 1 reports details on the meshes used for the numerical experiments. All simulations were performed on the LEONARDO supercomputer made available by CINECA, Italy.^3^

**TABLE 1:** Number of elements, nodes, and minimum, average and maximum element diameter for the meshes used in this work. The sphere and cylinder meshes were generated using gmsh [57], while the heart meshes were generated with VMTK [58] and the techinques discussed in Reference [59].

Test case	Mesh	Element type	# Elements	# Nodes	$h_{\min}$ [mm]	$h_{\mathrm{avg}}$ [mm]	$h_{\max}$ [mm]
Sphere (Section 3.1)	Fluid	Tetrahedra	1,154,477	196,239	0.492	0.829	1.140
	Solid	Tetrahedra	1,171,822	218,394	0.481	0.816	1.137
Cylinder (Section 3.2)	Fluid	Hexahedra	64,000	68,670	1.186	1.402	1.766
	Solid	Hexahedra	48,000	56,448	1.348	1.426	1.506
Heart (Section 3.3)	Fluid	Tetrahedra	1,306,215	225,801	0.513	1.487	4.031
	Solid	Tetrahedra	790,901	158,877	0.341	1.343	3.241

### 3.1 Idealized Spherical Benchmark

**FIGURE 2:**
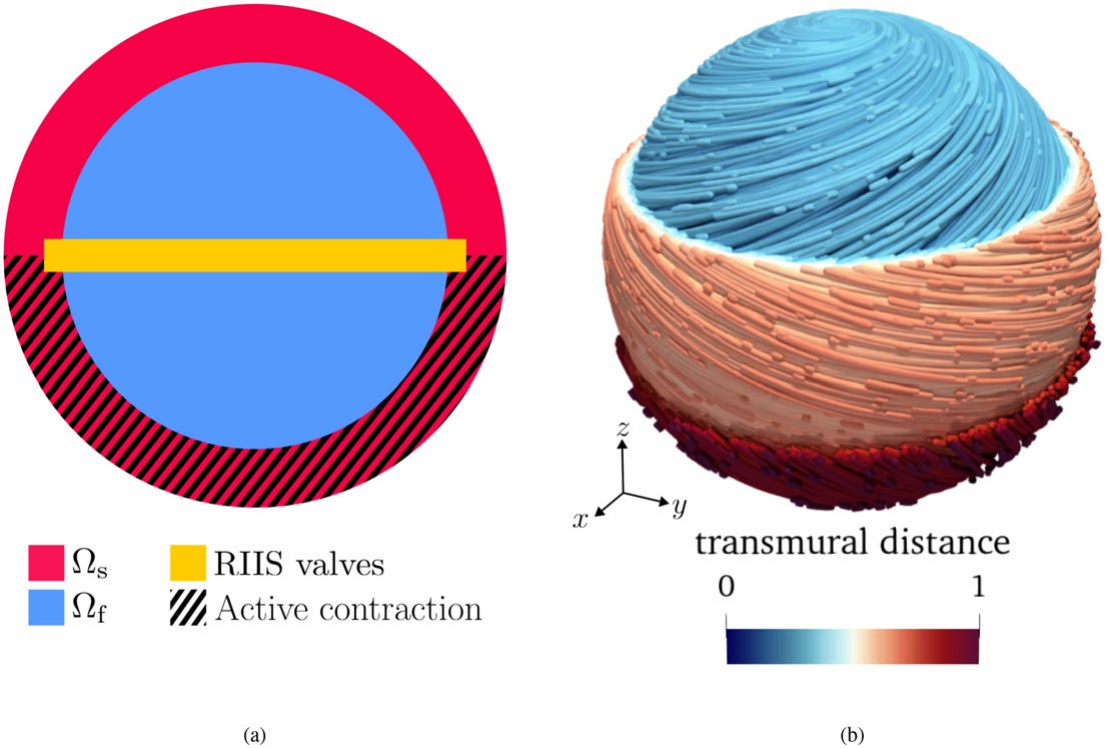
Idealized spherical benchmark (Section 3.1). (a) Schematic representation of the domain. (b) Streamline representation of the fiber field, colored according to the transmural distance. We show clipped layers of the solid domain for clarity.

We consider a geometrically idealized setting in which the fluid domain is a sphere, the structure domain is a spherical shell surrounding it, and a resistive planar surface cuts the domain in half, as depicted in Figure 2a. We assume that the solid follows the transversely isotropic Guccione constitutive law for ventricular tissue [41], with the same coefficients as in Reference [42]. We prescribe a fiber distribution (Figure 2b) that is tangent to the sphere, with transmurally varying orientation, inspired by ventricular fiber generation methods [60]. We prescribe an active contraction in the bottom half of the sphere, with the following expression:

$$\begin{array}{c} P_{s}^{\mathrm{act}}\left(\hat{d},t\right)=\left\{\begin{array}{cc} \frac{A_{\max}}{2}\left(1-\cos\left(\frac{\mathrm{\pi t}}{T_{\max}}\right)\right)\frac{\hat{F}\hat{f}⊗\hat{f}}{∥\hat{F}\hat{f}∥} & \text{if}\;z\leq 0,\\ \;0 & \text{otherwise}, \end{array}\right. \end{array}$$

where $\hat{f}$ is the orientation of the fiber field in the undeformed configuration, $A_{\max}=5\;\mathrm{kPa}$ is the peak contraction, and $T_{\max}=0.25\;s$ is the time at which that peak occurs. With this choice, the structure contracts in the bottom half, leading to an increase in pressure in the lower chamber, which mimics in this idealized setting the behavior of a ventricle and atrium during ventricular systole. We assume the system to be initially at rest, that is, u=0, $\hat{d}=0$ and $\frac{\partial \hat{d}}{\partial t}=0$ at time t=0. We impose homogeneous Neumann boundary conditions on the whole external boundary of the solid, that is

$$P_{s}\left(\hat{d},t\right)n=0\;\mathrm{on}\;\partial {\hat{\Omega }}_{s}\backslash \hat{\Sigma }.$$

No boundary conditions are needed for the fluid subproblem, since the fluid domain boundary coincides with the fluid–solid interface.

Under these conditions, we expect that the center of mass of the fluid–solid system, initially placed in the origin of the reference system, does not move. Indeed, there are no external forces acting on the system, and the prescribed active contraction gives rise to internal forces whose resultant is null, by Newton's third law. The surface cutting the domain in half prevents flow between the upper and lower parts of the fluid domain. Therefore, we expect the pressure to rise in the bottom chamber, due to the contraction, but without any average displacement of the fluid–solid system.

We use a harmonic extension operator to displace the fluid domain [40]. We set the final time to T=0.25s and, unless otherwise specified, we use a time step size $\Delta t=5\cdot 10^{-4}\;s$. We run simulations in this setting both with and withouth the newly introduced term $\hat{g}$.

**FIGURE 3:**
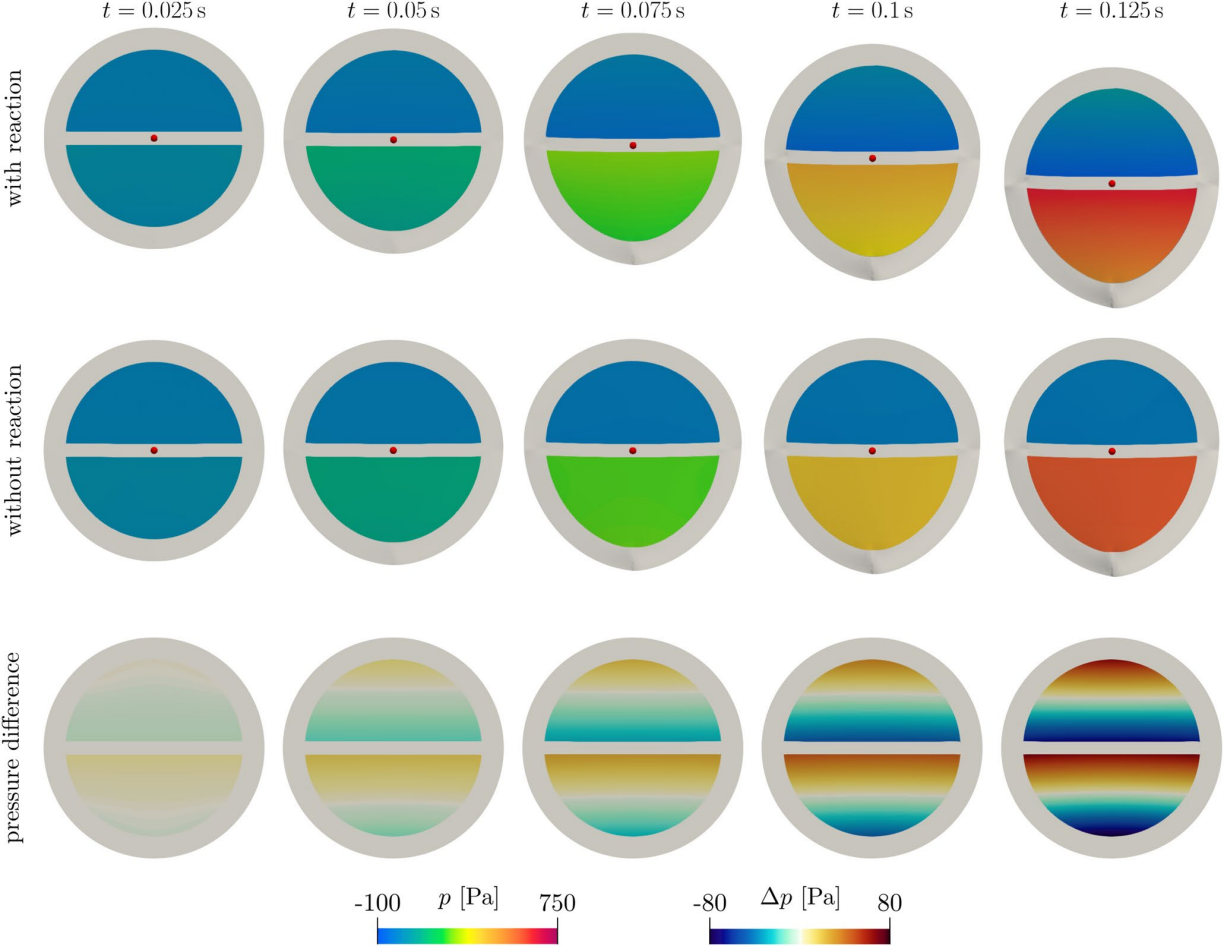
Idealized spherical benchmark (Section 3.1). Top and middle row: Evolution over time of the system deformation and fluid pressure, without (top) and with (middle) the force g. The red dot marks the position of the system's center of mass. Bottom row: Pressure difference between the simulations without and with the force g, represented on the reference configuration $\hat{\Omega }$.

**FIGURE 4:**
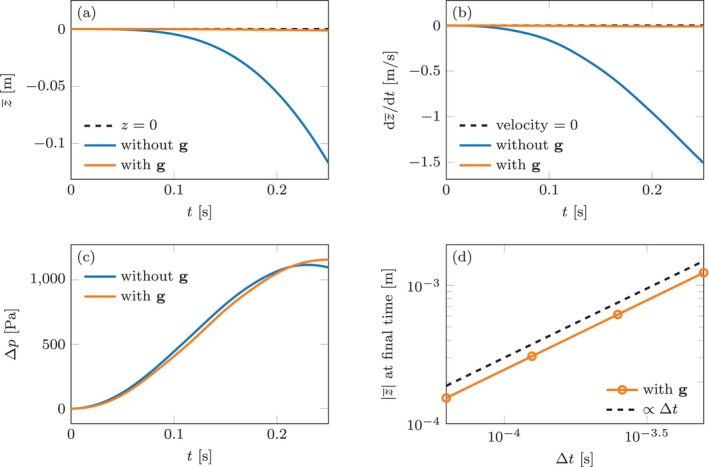
Idealized spherical benchmark (Section 3.1). Unless otherwise stated, the plots are obtained with $\Delta t=5\cdot 10^{-4}s$. (a) Vertical position $\bar{z}$ of the center of mass of the FSI system over time, with and without the attachment force g. (b) Vertical velocity of the center of mass over time, with and without the attachment force g. (c) Pressure jump over time between the lower and upper chambers of the sphere, with and without the attachment force g. (d) Vertical position of the center of mass of the FSI system at the final simulation time, plotted against the time step size Δt. We omit the plot for the case that neglects g, for which the final vertical position is approximately $|\bar{z}|\;=1.5\cdot 10^{-1}m$ for all choices of Δt.

Figure 3 reports some snapshots of the solution. Figure 4a,b plot the evolution of the vertical position (denoted by $\bar{z}$) and velocity of the center of mass of the whole fluid–solid system in deformed configuration, denoted by $\bar{x}$. When the forces $\hat{g}$ are not included, the center of mass exhibits a clear downward acceleration, which is unphysical. As discussed in Section 2.2, this happens due to the higher pressure in the bottom chamber not being properly balanced (Figure 4c), and thus accelerating the system downwards.

Conversely, including $\hat{g}$ in the solid momentum equation removes this imbalance, thus causing the center of mass to remain stationary. A small downward drift is still observed (Figure 4a), which is due to the explicit time discretization used for $\hat{g}$. Indeed, as the time step size Δt is reduced, the spurious drift tends to zero, with approximate order 1, which is consistent with the order of the explicit discretization. We verify this by plotting the vertical position of the center of mass at the final simulation time T with different choices of Δt in Figure 4d.

**FIGURE 5:**
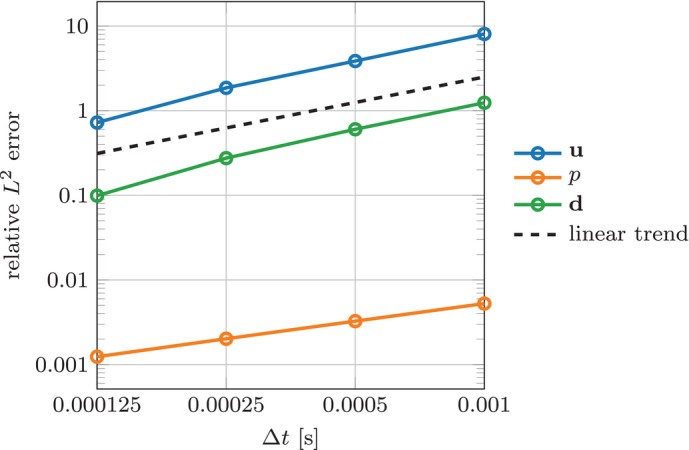
Idealized spherical benchmark (Section 3.1). Convergence with respect to the time step Δt of the $L^{2}$ error against a reference solution computed with $\Delta t=6.25\cdot 10^{-5}\;s$. The errors are relative to the norm of the reference solution.

We study convergence of the solution with respect to the time discretization step Δt. In absence of an analytical solution, we consider the numerical solution obtained with $\Delta t=6.25\cdot 10^{-5}\;s$ as a reference, and compute the relative $L^{2}$ error in the velocity u, pressure p and displacement d variables with varying choices of Δt. The results are reported in Figure 5. We observe linear convergence for u and d, as expected. The observed convergence order for p is approximately 0.7, which we attribute to a saturation of the error associated to spatial convergence, seeing as the mesh size is kept fixed.

Table 2 reports a breakdown of the computational cost in the two cases. The computational cost when they are included is only marginally larger than when they are neglected, thanks to the choice of computing the valve forces explicitly. The cost is dominated by the solution of the linear system arising from the discretization of the FSI problem, with the computation of $\hat{g}$ being a minor additional cost.

**TABLE 2:** Idealized spherical benchmark (Section 3.1). Breakdown of the simulation wall time, both with and without valve attachment forces g.

	Without g [s]	With g [s]
Total time	1693.3	1787.3
Linear solver	1518.9	1554.3
Fluid assembly	293.6	299.9
Solid assembly	219.6	223.1
Compute g	—	88.5

### 3.2 Idealized Cylindrical Benchmark

**FIGURE 6:**
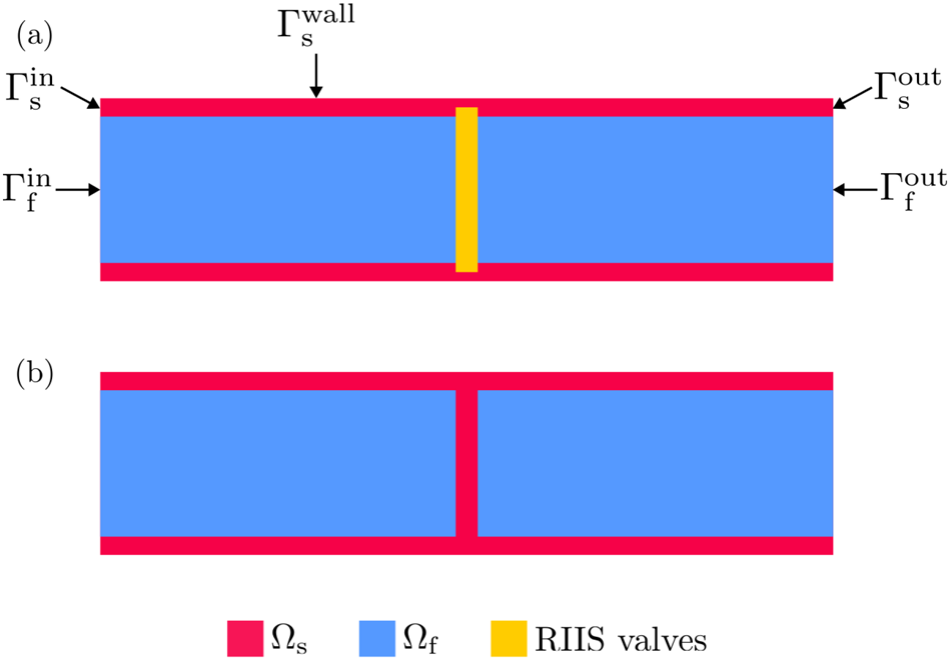
Schematic representation of the domain for the idealized cylindrical benchmark (Section 3.2). (a) Model in which the valve is represented through RIIS. (b) Model in which the valve is represented as a deformable solid.

We now consider the domain depicted in Figure 6a, consisting of a cylindrical fluid domain enclosed by a solid cylindrical shell, divided in half by a planar surface perpendicular to the axis of the cylinder. While the idealized scenario of Section 3.1 mimics the interaction between the left ventricle and left atrium during systole, that is, with a closed mitral valve (MV), this test case mimics the interaction of a left ventricle and the ascending aorta during diastole, that is, with a closed aortic valve (AV).

No part of the solid domain undergoes active contraction in this test. We consider a neo‐Hookean constitutive law [15, 42], with the following stress tensor:

(5) $$P_{s}^{\mathrm{pas}}\left(d\right)=\mu_{s}J^{-\frac{2}{3}}\left(F-\frac{1}{3}\left(F:F\right)F^{-⊤}\right)+\frac{\kappa_{s}}{2}\left(J\left(J-1\right)+\log J\right)F^{-⊤}.$$

We set $\mu_{s}=10^{5}\mathrm{Pa}$ and $\kappa_{s}=5\cdot 10^{4}\mathrm{Pa}$.

The terminal sections of the solid subdomain are kept fixed (i.e., d=0 on $\Gamma_{s}^{\text{in}}$ and $\Gamma_{s}^{\text{out}}$), while on the outer wall $\Gamma_{s}^{\text{wall}}$ we impose the following spring‐dashpot condition [42]:

$$P_{s}\left(d\right)n=-K\left(n⊗n\right)d-C\frac{\partial d}{\partial t},$$

where $K=2\cdot 10^{8}\mathrm{Pa}/m$ is a normal boundary stiffness coefficient, and $C=2\cdot 10^{4}\mathrm{Pa}\cdot s/m$ is a boundary viscosity coefficient. At the inlet and outlet boundaries of the fluid domain, we impose Neumann conditions:

$$\begin{array}{r} \sigma_{f}\left(u,p\right)n=-p_{\text{in}}\left(t\right)n\;\mathrm{on}\;\Gamma_{f}^{\text{in}},\\ \sigma_{f}\left(u,p\right)n=0\;\mathrm{on}\;\Gamma_{f}^{\text{out}}. \end{array}$$

The inlet pressure $p_{\text{in}}$ gradually increases from $p_{\text{in}}^{\min}=0\;\mathrm{Pa}$ to $p_{\text{in}}^{\max}=10^{4}\;\mathrm{Pa}$ during the initial $T_{\text{load}}=0.25\;s$ of simulation, according to the following expression:

$$\begin{array}{c} p_{\text{in}}\left(t\right)=\left\{\begin{array}{cc} p_{\text{in}}^{\min}+\frac{1}{2}\left(p_{\text{in}}^{\max}-p_{\text{in}}^{\min}\right)\left(1-\cos\left(\frac{\mathrm{\pi t}}{T_{\text{load}}}\right)\right) & \text{for}\;0\leq t<T_{\text{load}},\\ \;p_{\text{in}}^{\max} & \text{for}\;t\geq T_{\text{load}}. \end{array}\right. \end{array}$$

We simulate this scenario using three models. In the first, we model the valve with RIIS, and include the force g. In the second, we neglect the force g. In the third scenario, the valve is not modeled with RIIS, but instead it is included in the solid domain ${\hat{\Omega }}_{s}$ (see Figure 6b), with neo Hooke constitutive law given by (5), setting $\mu_{s}=5\cdot 10^{7}\mathrm{Pa}$ and $\kappa_{s}=5\cdot 10^{5}\mathrm{Pa}$. This third scenario, in which we effectively simulate FSI on the surface, will be regarded as the ground truth to assess whether the resistive approximation of the two other settings is physically accurate.

Under these conditions, we expect that, as the inlet pressure increases, the pressure gradient through the valve results in a net force acting on it, pushing it towards the outlet, together with the walls to which it is attached. Accordingly, we expect that the valve displaces towards the outlet.

**FIGURE 7:**
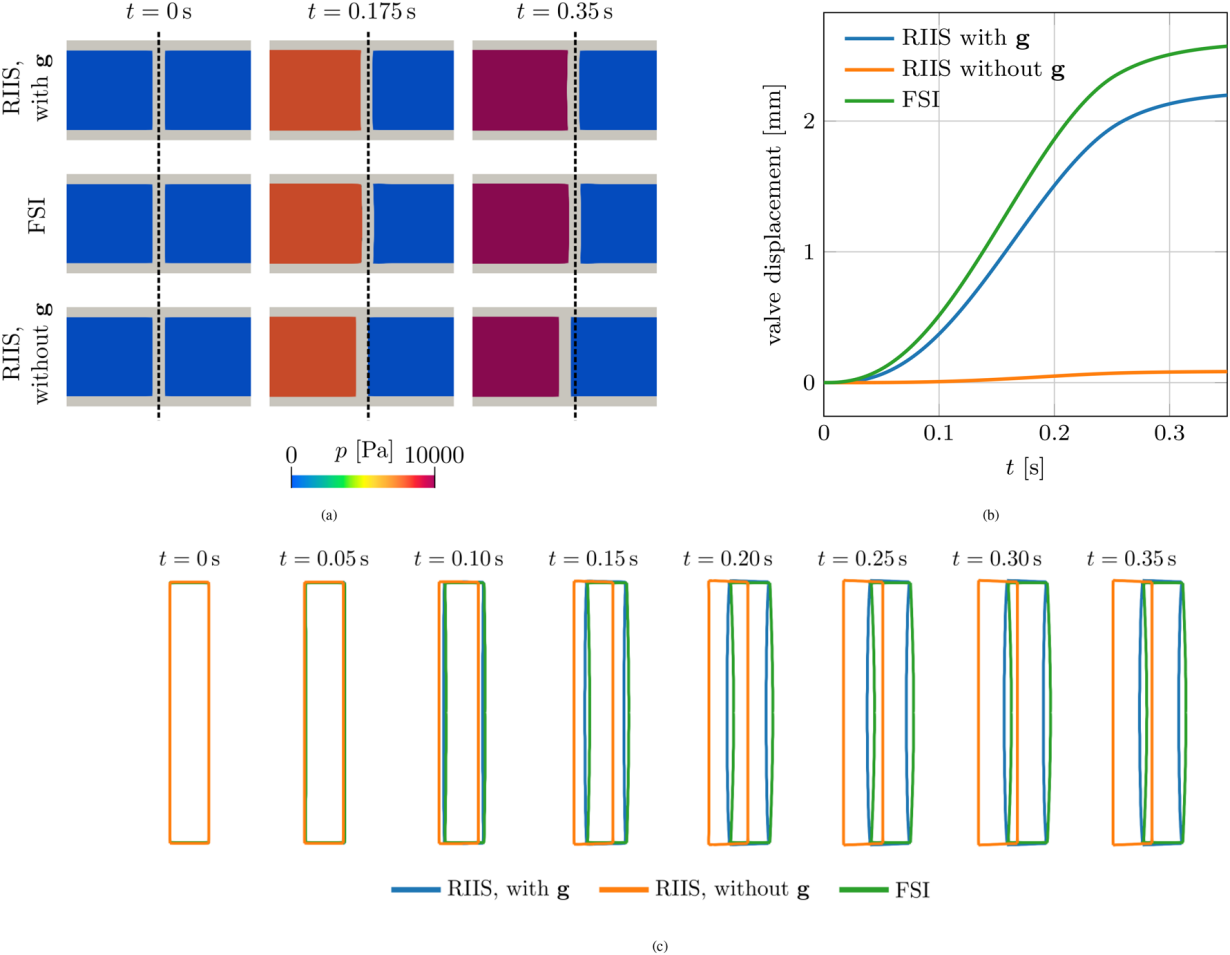
Idealized cylindrical benchmark (Section 3.2). (a) Pressure inside the cylinder and displacement of the valve at three different time instants, with the three different models under consideration. From top to bottom: RIIS model including the force g, ground‐truth FSI model and RIIS model without g. The dashed line marks the position of the center of the valve in the FSI model. Notice that the domain is cropped to focus on the valve. (b) Average displacement of the valve with the three models considered. (c) Outline of the valve's longitudinal section computed with the three models.

Figure 7a reports some snapshots of the solution with the three models, while Figure 7b plots the evolution of the longitudinal displacement of the valve in response to the increase in inlet pressure, and Figure 7c highlights the valve displacement and deformation. The ground‐truth model, using FSI for the valve, yields results consistent with the expectations: as the inlet pressure increases, the valve is pushed towards the outlet. The model with RIIS and including the force g yields a valve displacement that is consistent with this behavior. While the valve deformation is different from that of the ground truth, due to the differences in the underlying displacement model (the ALE displacement in the resistive model, the neo‐Hooke law in the ground‐truth model), the trend and overall displacement are the same. Conversely, in the RIIS model without g we observe a very small valve displacement, and the results are qualitatively very different from the ground truth setting. Indeed, in the model including g, the pressure gradient acting on the valve leads to a net force on the wall, pushing it towards the outlet. When the wall displaces in response to this force, the fluid domain follows it, and so does the valve. Conversely, when neglecting g, the model does not include the force that pushes on the valve due to the pressure gradient, and there is no displacement in response to that force.

This test indicates that the newly proposed force g causes the RIIS approach to yield results that are in closer agreement with standard body‐fitted FSI models of the valve, thereby improving the physical accuracy of the resistive method.

### 3.3 Realistic Left Heart Model

**FIGURE 8:**
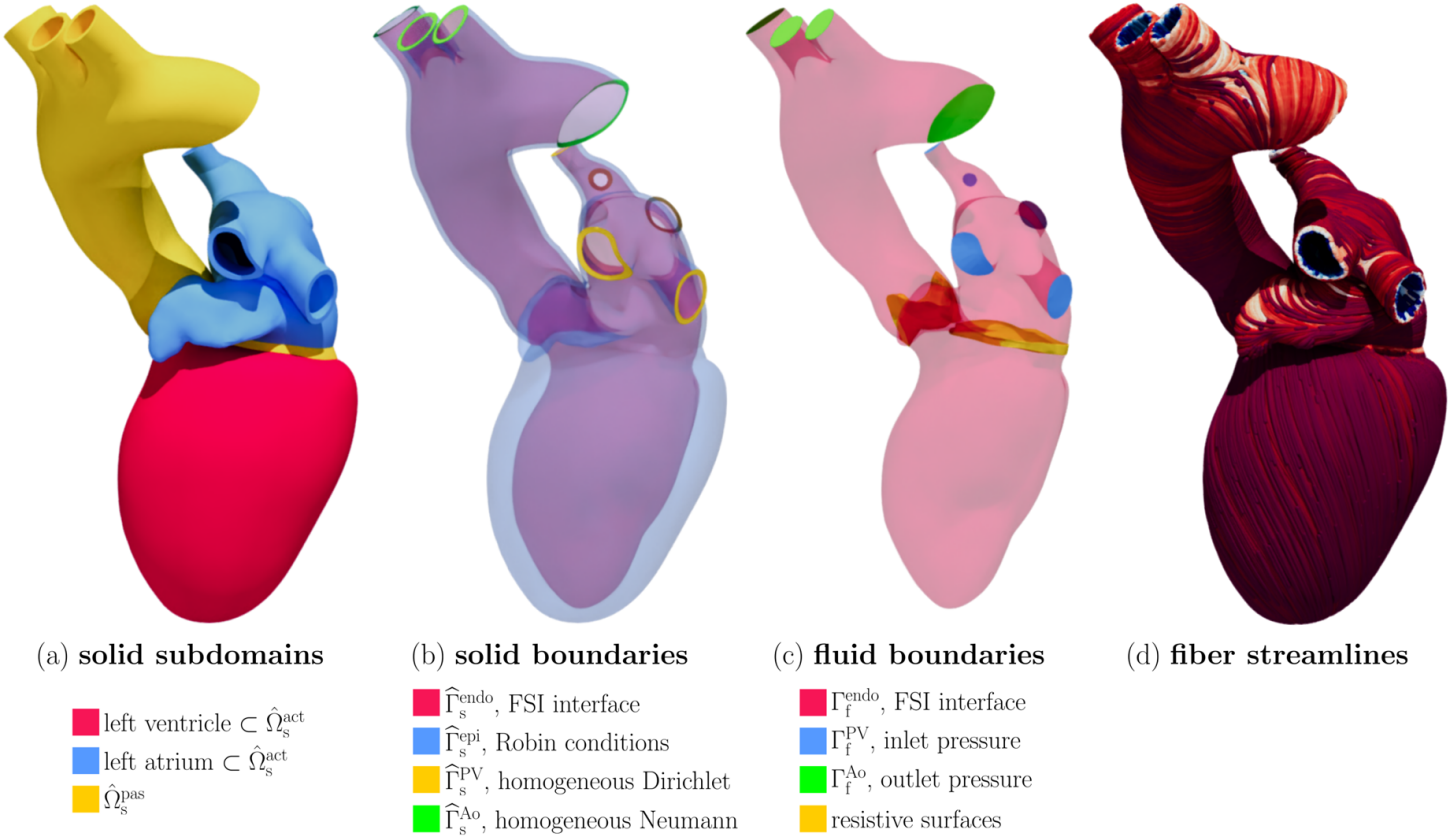
Left heart test case. (a) Decomposition of the solid domain ${\hat{\Omega }}_{s}$ into the active part ${\hat{\Omega }}_{s}^{\mathrm{act}}$, including the ventricle and atrium, and the passive part ${\hat{\Omega }}_{s}^{\mathrm{pas}}$, including the aorta and atrioventricular ring. (b) Solid domain boundaries. (c) Fluid domain boundaries. (d) Streamlines of the fiber field f, colored according to the transmural coordinate for readability.

We consider a cardiac FSI model driven by electrophysiology, adapted from Reference [15]. The domain, depicted in Figure 8, represents a realistic human left heart [61]. With respect to the original publication [15], we replace the monodomain model of cardiac electrophysiology with an eikonal‐diffusion model [44], in the interest of computational efficiency. Additionally, we include atrial contraction, which was previously neglected. The model is described in more detail in Appendix A. We set the heartbeat period to $T_{\mathrm{hb}}=0.8\;s$.

**FIGURE A1:**
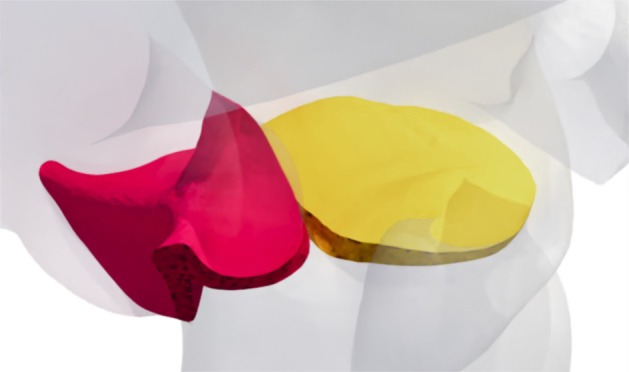
Left heart test case. Closeup visualization of the AV (red) and MV (yellow) in their closed configuration at the beginning of the simulation.

The model includes two resistive surfaces, to represent the MV and AV (Figure A1). As done in References [15, 24], the valves open and close according to the pressure difference between upstream and downstream.

**FIGURE 9:**
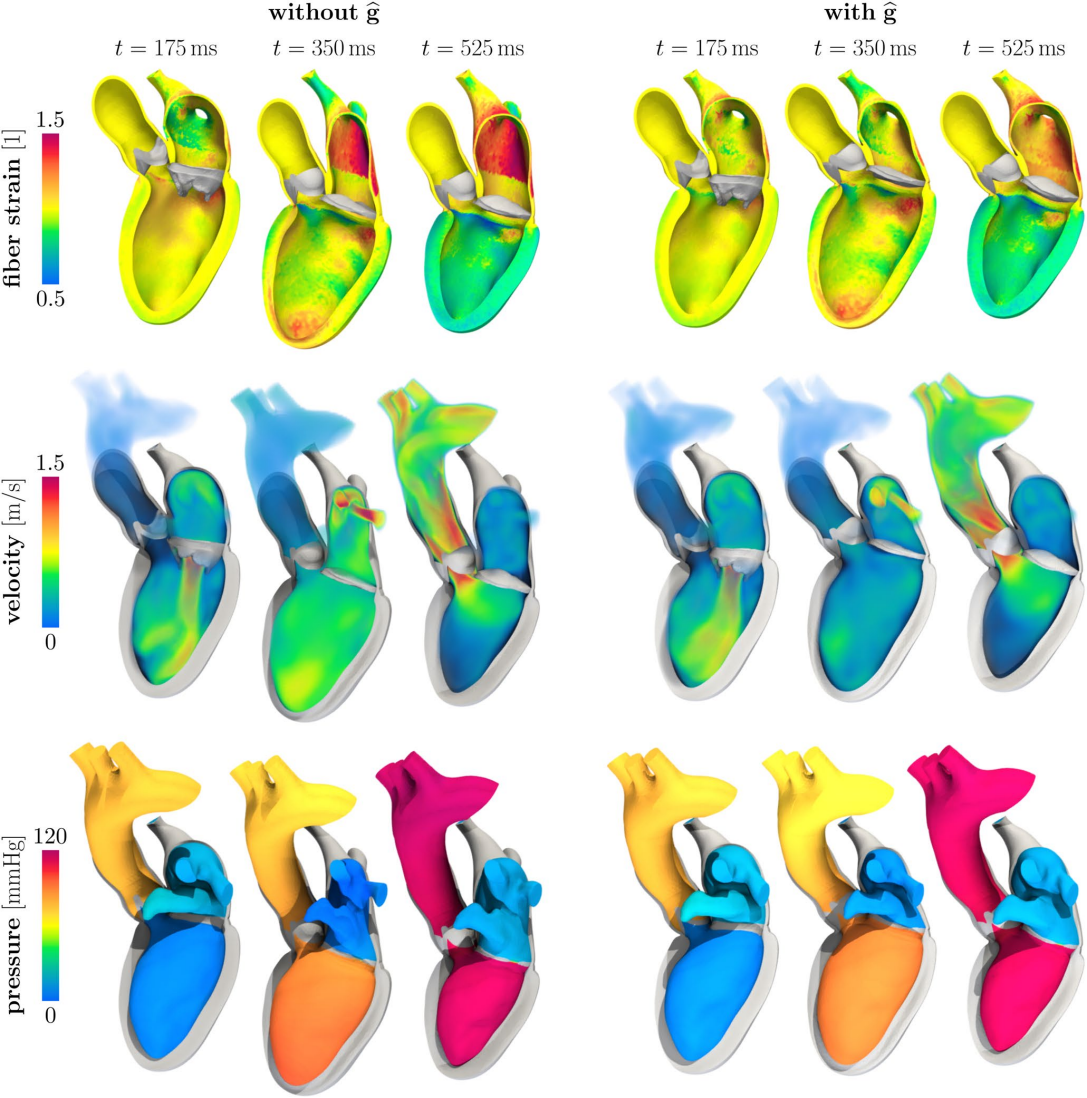
Left heart test case. Snapshots of the numerical solution without (left) and with (right) the valve forces $\hat{g}$. We report the strain along fibers $∥\hat{F}\hat{f}∥$ (top), a volume rendering of the fluid velocity u (middle) and the pressure p (bottom). The domain is represented in the deformed configuration. For each, we report a frame during diastolic filling (t=175ms), one during isovolumetric contraction (t=350ms) and one during ejection (t=525ms).

**FIGURE 10:**
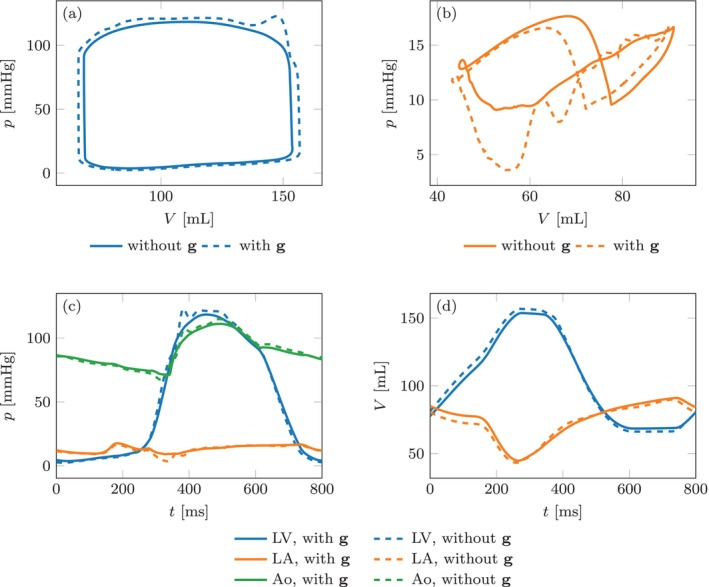
Left heart test case. (a) Left ventricular pressure‐volume loop. (b) Left atrial pressure‐volume loop. (c) Traces of pressure in the ventricle (LV), atrium (LA) and ascending aorta (Ao) over time. (d) Traces of volume of the ventricular and atrial chamber over time. In all the plots, the solid lines correspond to the simulation including valve forces, and the dashed lines to the simulation neglecting them.

We use a time step size $\Delta t=2\;\cdot \;10^{-4}\;s$. The simulation runs for two heart cycles (T=1.6s), and we report results on the second one, for which the simulation has approximately reached a periodic state in terms of chamber pressures and volumes. The times reported in this section are relative to the second heartbeat. Figure 9 displays some snapshots of the numerical solution, and Figure 10 reports the traces of pressure and volume in the chambers, as well as the evolution of the force densities $\frac{F_{k}}{V_{k}}$ for the two valves.

The cardiac cycle starts during ventricular diastole, with AV closed and MV open. The atrium contracts around time t=100ms, which is shortly followed by ventricular contraction. This causes the ventricular pressure to rise and the MV to close, beginning isovolumetric contraction.

If we neglect the valve forces, the high ventricular pressure leads to a force that pulls the ventricle in the direction of the apex (as schematized in Figure 1, and visualized in Figure 9). This results in a fast and large displacement of the ventricle along its longitudinal axis, which also gives rise to high strains in the atrial wall (Figure 9). Correspondingly, there are strong jets in the pulmonary veins, which are absent if the valve forces are included in the model. This behavior becomes apparent also in pressure and volume traces and in the pressure‐volume loops of both the atrium and ventricle (Figure 10), which show sharp oscillations at the beginning of the systolic phase. During diastole, due to the atrial and ventricular pressures being approximately equal and the MV being open, the elasticity of the atrium and the pericardial boundary conditions allow the solid to return to its original configuration. As previously discussed, this unphysical behavior arises from an incorrect balance of forces in the FSI system, which is most evident when there are significant differences among the chamber pressures, such as during ventricular systole.

We further remark that the high strains may lead to an unrealistic feedback effect on the force generation model [43], which is sensitive to both fiber strain and strain rate. Additionally, in more sophisticated models that include detailed mechano‐electric feedbacks, such deformations may even trigger abnormal electrical activity [62], thus largely affecting the simulation outcome.

If instead we include the valve forces, we no longer observe any unphysical oscillations in the ventricular displacement, nor in the pressure–volume loops. This is evident in particular in the morphology of the atrial loop, which is much more consistent with physiology if valve forces are included in the model [42, 63, 64].

**FIGURE 11:**
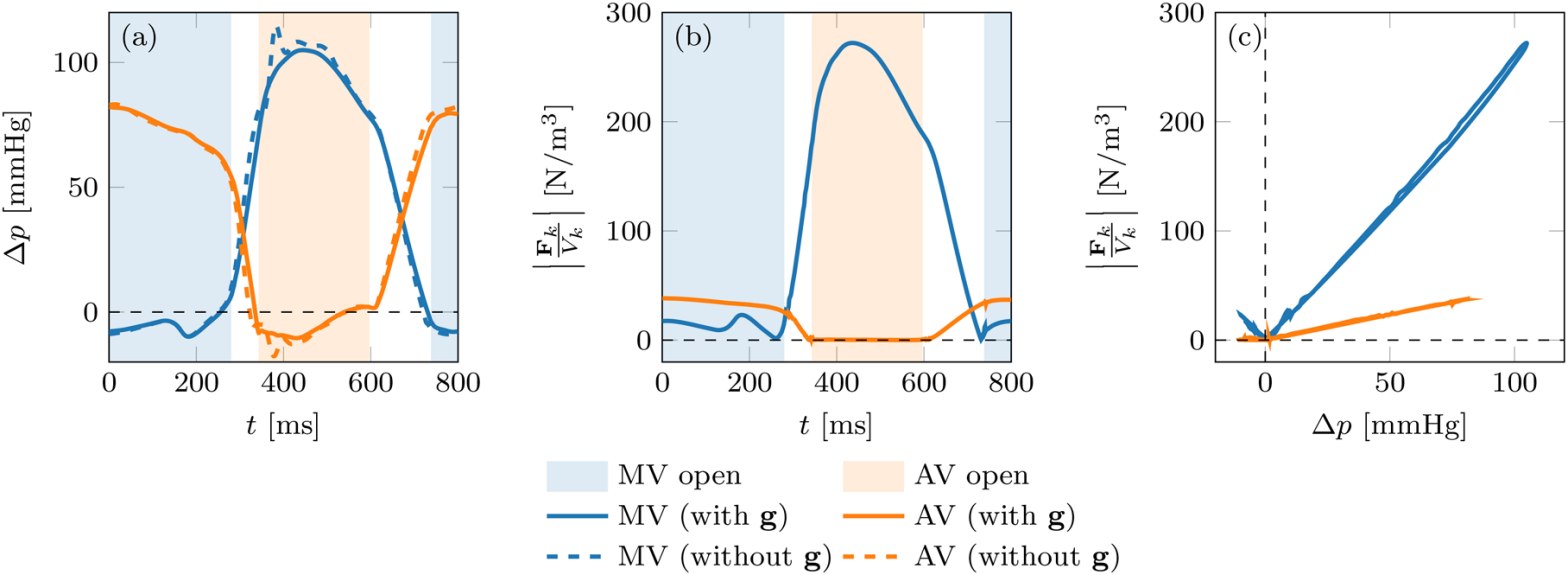
Left heart test case. (a) Pressure jump between downstream and upstream over time for each valve, that is, $\Delta p=p_{\text{down}}-p_{\mathrm{up}}$. (b) Magnitude of the force density $\frac{F_{k}}{V_{k}}$ over time for the two valves. (c) Magnitude of the force density plotted against the pressure jump for the two valves. Shaded areas in plots (a) and (b) highlight times during which one of the valves is open.

Figure 11 reports the evolution of the pressure jump between downstream and upstream and of valve force densities $\frac{F_{k}}{V_{k}}$ for the two valves. We observe that the pressure jumps with and without g exhibit differences consistent with those shown in Figure 10. While the overall evolution of pressure is similar in the two cases, we remark that the two scenarios exhibit markedly different displacements and strains, as seen in Figure 9.

Considering only the closed valve configuration, corresponding to positive pressure jumps, Figure 11 highlights a nearly perfect linear relation between pressure jump and force. The slope of this relation is significantly higher for the MV ($2.5\;N/\left(m^{3}\cdot \text{mmHg}\right)$) than it is for the AV ($0.47\;N/\left(m^{3}\cdot \text{mmHg}\right)$). We attribute this effect to the different surface areas, curvatures and overall geometry of the two valves in closed configuration (Figure A1).

## 4 Conclusions

This paper introduces an improvement of the RIIS model, allowing it to represent valve leaflets in FSI simulations of the heart, by accounting for the attachment forces exchanged by valves and cardiac walls. These forces are computed in such a way as to balance the forces exchanged by the fluid and the valves, thus making sure that the model satisfies Newton's laws. Numerical experiments illustrate the issues arising from a naive application of RIIS in FSI contexts, as well as the improvements obtained by including the proposed modification. A test case using a realistic left heart model highlights how including valve attachment forces is crucial to obtaining a physically meaningful behavior of the heart. While the present work focuses on RIIS, the same principles may be straightforwardly extended to other resistive methods for valve modeling, thus providing a general approach to include simplified models of valves in FSI simulations of the heart.

## Funding

This work was supported by HORIZON EUROPE Digital, Industry and Space, 101172493.

## Ethics Statement

The authors have nothing to report.

## Conflicts of Interest

The authors declare no conflicts of interest.

## Data Availability

Data for the geometrically spherical and cylindrical test cases can be made available upon request. Data for the left heart test cannot be made available due to commercial restrictions on the model geometry.

## Endnotes

^1^

https://lifex.gitlab.io.

^2^

https://dealii.org.

^3^
Technical specifications: https://wiki.u‐gov.it/confluence/display/SCAIUS/UG3.2%3A+LEONARDO+UserGuide.

## Appendix A Left Heart EIKONAL‐Mechanics‐Fluid Model

This section describes the cardiac electro‐fluid‐mechanics model used in the numerical experiments of Section 3.3. The model is based on the one presented in Reference [15], which in turn combines electromechanical and fluid models that were also discussed in References [24, 42]. We refer to the original publications for a comparison and validation of the simulation results against reference data from the medical literature.

We partition the solid domain into an electrically active part ${\hat{\Omega }}_{s}^{\mathrm{act}}$ and a passive part ${\hat{\Omega }}_{s}^{\mathrm{pas}}$, such that ${\hat{\Omega }}_{s}={\hat{\Omega }}_{s}^{\mathrm{act}}\cup {\hat{\Omega }}_{s}^{\mathrm{pas}}$. The active part includes the left ventricle and left atrium, whereas the passive part includes the atrioventricular ring and the ascending aorta. Additionally, we denote by ${\hat{\Gamma }}_{s}^{\mathrm{epi}}$ the epicardial boundary of the structure subdomain, by ${\hat{\Gamma }}_{s}^{\mathrm{PV}}$ the pulmonary vein inlet sections, and by ${\hat{\Gamma }}_{s}^{\mathrm{Ao}}$ the aorta outlet sections. Similarly, ${\hat{\Gamma }}_{f}^{\mathrm{PV}}$ and ${\hat{\Gamma }}_{f}^{\mathrm{Ao}}$ denote the inlet and outlet boundaries of the fluid subdomain.

Fibers are generated by adapting the method presented in Reference [42], by restricting the whole‐heart fibers to the left heart geometry considered here. This defines an orthonormal triplet $\left\{\hat{f},\hat{s},\hat{n}\right\}$ at every point of the domain [60].

We define the contraction of the muscle through the eikonal‐mechanics framework previously described in Reference [44]. To this end, prior to the FSI simulation, we solve the following eikonal‐diffusion problem in the active solid subdomain:

(A1)
$$\begin{array}{c} \left\{\begin{array}{cc} c_{0}\sqrt{\text{∇}\varphi \cdot D\text{∇}\varphi }-\text{∇}\cdot \left(D\text{∇}\varphi \right)=1 & \text{in}\;{\hat{\Omega }}_{s}^{\mathrm{act}},\\ \;\varphi =\varphi_{0} & \text{in}\;\hat{S}\subset {\hat{\Omega }}_{s}^{\mathrm{act}},\\ \;\left(\Sigma \text{∇}\varphi \right)\cdot n=0 & \mathrm{on}\;\partial {\hat{\Omega }}_{s}^{\mathrm{act}}\backslash \hat{S}, \end{array}\right. \end{array}$$

where $\varphi :{\hat{\Omega }}_{s}^{\mathrm{act}}\to \left[0,T_{\mathrm{hb}}\right)$ is the activation time, that is the time at which points in the active subdomain are reached by the excitation front, and $T_{\mathrm{hb}}>0$ is the heartbeat period. D is a diffusion tensor, defined by

$$D=\sigma_{f}\hat{f}⊗\hat{f}+\sigma_{s}\hat{s}⊗\hat{s}+\sigma_{n}\hat{n}⊗\hat{n},$$

where $\sigma_{f}$, $\sigma_{s}$ and $\sigma_{n}$ are the positive conductivity coefficients along the directions of fibers, sheets and normals. Following Reference [44], we solve problem (A1) through a pseudo‐time algorithm. Notice that by solving (A1) only on the active subdomain ${\hat{\Omega }}_{s}^{\mathrm{act}}$, we model the fact that the passive subdomain acts as an electrical insulant between atrium and ventricle [42]. The parameters for the eikonal‐diffusion model are reported in Table A1.

Independently, we solve a system of ordinary differential equations (ODEs) representing the ionic activity of a single cell:

$$\left\{\begin{array}{cc} \frac{dw}{dt}=F_{\mathrm{ion}}\left(w,u\right) & \text{in}\;\left(0,T\right)\;,\\ \frac{\mathrm{du}}{dt}+I_{\mathrm{ion}}\left(w,u\right)=I_{\mathrm{app}}\left(t\right) & \text{in}\;\left(0,T\right)\;,\\ \;w\left(0\right)=w_{0}\;, & \end{array}\right.$$

where $w:\left[0,T\right)\to {\mathbb{R}}^{N_{\mathrm{ion}}}$ is a vector of ionic state variables, the functions $F_{\mathrm{ion}}$ and $I_{\mathrm{ion}}$ depend on the specific ionic model being considered, and $I_{\mathrm{app}}$ is a prescribed stimulus current triggering the excitation. We solve the ionic model for 1000 heartbeat cycles, and then extract from the state vector w the component representing the evolution of the intracellular calcium concentration at the last cycle, denoted by ${\left[{\mathrm{Ca}}^{2+}\right]}_{i}^{0}\left(t\right):\left[0,T_{\mathrm{hb}}\right)\to \mathbb{R}$. We consider the model by ten Tusscher and Panfilov [65] for ventricular tissue and the one by Courtemanche, Ramirez and Nattel [66] for atrial tissue. Finally, we combine the activation time and the calcium transient to compute the evolution of intracellular calcium at every point of the domain:

$${\left[{\mathrm{Ca}}^{2+}\right]}_{i}\left(x,t\right)={\left[{\mathrm{Ca}}^{2+}\right]}_{i}^{0}\left(\left(t\mathrm{mod}T_{\mathrm{hb}}\right)-\varphi \left(x\right)\right)\;x\in {\hat{\Omega }}_{s}^{\mathrm{act}},\;t\in \left(0,T\right),$$

where amodb denotes the remainder of the division between a and b, that is $a\;\mathrm{mod}\;b=a-b\left\lfloor \frac{a}{b}\right\rfloor$. In other words, the calcium transient is repeated periodically at every point, with period $T_{\mathrm{hb}}$, and shifted by the activation time φ.

The generation of contractile force in the muscle is regulated by the RDQ20‐MF model [24, 42, 43], which is expressed by the following system

$$\frac{\partial s}{\partial t}=F_{\mathrm{act}}\left({\left[{\mathrm{Ca}}^{2+}\right]}_{i},\mathrm{SL},\frac{\mathrm{\partial SL}}{\partial t}\right)\;\text{in}\;{\hat{\Omega }}_{s}^{\mathrm{act}}\times \left(0,T\right),$$

where s is the activation state vector, $F_{\mathrm{act}}$ is the forcing term, defined as in Reference [43], and $\mathrm{SL}={\mathrm{SL}}_{0}∥\hat{F}\hat{f}∥$ is the sarcomere length. Table A2 reports the values of the RDQ20‐MF model parameters. The active stress tensor is then defined as

$$P_{s}^{\mathrm{act}}\left(\hat{d},t\right)=T^{\mathrm{act}}\left(s\right)\left(n_{f}\frac{\hat{F}\hat{f}⊗\hat{f}}{∥\hat{F}\hat{f}∥}+n_{n}\frac{\hat{F}\hat{n}⊗\hat{n}}{∥\hat{F}\hat{f}∥}\right),$$

with $T^{\mathrm{act}}$ defined as in Reference [43]. The coefficients $n_{f}$ and $n_{s}$ scale the amount of contraction along fibers and normals. In the passive subdomain ${\hat{\Omega }}_{s}^{\mathrm{pas}}$, the active stress tensor is set to zero.

Concerning passive stress, we use the Guccione model for atrial and ventricular tissue [41], and a neo Hooke model for the atrioventricular ring and for the aortic wall, as done in References [15, 42]. The interface between different material models is smoothed as discussed in Reference [15]. The evolution of wall displacement is regulated by the elastodynamics Equation (4). We impose homogeneous Dirichlet boundary conditions on the pulmonary vein sections ${\hat{\Gamma }}_{s}^{\mathrm{PV}}$, homogeneous Neumann conditions on the aortic sections ${\hat{\Gamma }}_{s}^{\mathrm{Ao}}$, and normal viscoelastic Robin conditions on the epicardial boundary ${\hat{\Gamma }}_{s}^{\mathrm{epi}}$ (Figure 8b), that is:

$$\begin{array}{c} \left\{\begin{array}{cc} d=0 & \mathrm{on}\;{\hat{\Gamma }}_{s}^{\mathrm{PV}},\\ \;P_{s}\left(d,t\right)n=0 & \mathrm{on}\;{\hat{\Gamma }}_{s}^{\mathrm{Ao}},\\ P_{s}\left(d,t\right)n=-\left(n⊗n\right)\left(K_{\mathrm{epi}}d+C_{\mathrm{epi}}\frac{\partial d}{\partial t}\right) & \mathrm{on}\;{\hat{\Gamma }}_{s}^{\mathrm{epi}}, \end{array}\right. \end{array}$$

where $K_{\mathrm{epi}}$ and $C_{\mathrm{epi}}$ are the positive stiffness and damping coefficients, respectively. Following Reference [42], we use two different sets of coefficients to distinguish between regions in contact with the pericardial sac and regions covered by epicardial fat. We report the parameters for the solid mechanics model in Table A4.

The fluid domain displacement is computed through a neo‐Hookean material law, that is

where $\mu_{\mathrm{ALE}}$ and $\kappa_{\mathrm{ALE}}$ are the stiffness and bulk modulus of the fictitious material representing the fluid domain.

Blood dynamics is modeled through Navier–Stokes Equations (1) in ALE reference. We include two resistive surfaces to represent the MV and AV. The displacements mapping the closed configuration onto the open one are derived through rule‐based procedures [16]. For each valve, the opening and closing are triggered by the pressure jump between upstream and downstream [15, 24]. Table A3 reports the parameters for the fluid model, including those for domain displacement and for RIIS.

At the inlet boundaries $\Gamma_{f}^{\mathrm{PV}}$ and at the outlet boundaries $\Gamma_{f}^{\mathrm{Ao}}$, the fluid model is coupled to a closed‐loop, lumped parameter model of the circulatory system [15, 24, 42]. The coupling is expressed by imposing pressure conditions at the inlet and outlet boundaries, where the pressure values are solutions to the circulation model. The flowrates measured at the inlet and outlet sections of the fluid dynamics model are then used as boundary conditions for the circulation model. The parameters for the circulation model are reported in Table A5.

For additional details on the model and on the numerical methods used for its solution, we refer to References [15, 36, 44], and.

**TABLE A1:** Left heart test case. Parameters for the eikonal‐diffusion model.

Subdomain	Parameter	Description	Value	Unit
All	$c_{0}$	Depolarization velocity	60	1/s^1/2^
Left ventricle	$\sigma_{f}$	Fiber conductivity	$1.00\cdot 10^{-4}$	m/s^2^
	$\sigma_{s}$	Sheet conductivity	$0.44\cdot 10^{-4}$	m/s^2^
	$\sigma_{n}$	Normal conductivity	$0.44\cdot 10^{-4}$	m/s^2^
Left atrium	$\sigma_{f}$	Fiber conductivity	$7.00\cdot 10^{-4}$	m/s^2^
	$\sigma_{s}$	Sheet conductivity	$1.41\cdot 10^{-4}$	m/s^2^
	$\sigma_{n}$	Normal conductivity	$1.41\cdot 10^{-4}$	m/s^2^

**TABLE A2:** Left heart test case. Parameters for the RDQ20‐MF force generation model. For the meaning of the parameters, we refer to the original publication. If not reported, the parameter values are those presented in the original paper (Reference [43], Table 3).

Subdomain	Parameter	Description	Value	Unit
Left ventricle	${\mathrm{SL}}_{0}$	Sarcomere slack length	2.2	μm
	$K_{d0}$	Dissociation constant	0.36	μM
	$\alpha_{\mathrm{Kd}}$	Dissociation constant sensitivity	−0.2083	μM/μm
	μ		10	—
	γ		30	—
	$K_{\mathrm{off}}$	Troponin dissociation rate	8	1/s
	$K_{\text{basic}}$	Tropomyosin reaction rate	4	1/s
	$r_{0}$	Crossbridge attachment‐detachment rate	134.31	1/s
	α	Crossbridge detachment rate sensitivity	25.184	—
	$\mu_{0,\mathrm{fP}}$	Zero‐th moment of attached rate	32.225	1/s
	$\mu_{1,\mathrm{fP}}$	First moment of attached rate	0.768	1/s
	$a_{\mathrm{XB}}$	Crossbridge stiffness	500	MPa
Left atrium	${\mathrm{SL}}_{0}$	Sarcomere slack length	1.9	μm
	$K_{d0}$	Dissociation constant	0.865	μM
	$\alpha_{\mathrm{Kd}}$	Dissociation constant sensitivity	−1.25	μM/μm
	μ		10	—
	γ		20	—
	$K_{\mathrm{off}}$	Troponin dissociation rate	180	1/s
	$K_{\text{basic}}$	Tropomyosin reaction rate	20	1/s
	$r_{0}$	Crossbridge attachment‐detachment rate	134.31	1/s
	α	Crossbridge detachment rate sensitivity	25.184	—
	$\mu_{0,\mathrm{fP}}$	Zero‐th moment of attached rate	32.235	1/s
	$\mu_{1,\mathrm{fP}}$	First moment of attached rate	0.768	1/s
	$a_{\mathrm{XB}}$	Crossbridge stiffness	200	MPa

**TABLE A3:** Left heart test case. Parameters for the fluid dynamics model. For the meaning of the parameters, we refer to Reference [24].

Submodel parameter	Description	Value	Unit	
Navier–Stokes	$\rho_{f}$	Fluid density	$1.06\cdot 10^{3}$	kg/m^3^
	μ	Fluid viscosity	$3.5\cdot 10^{-3}$	Pa·s
RIIS	$\varepsilon_{\mathrm{AV}}$, $\varepsilon_{\mathrm{MV}}$	Valve half‐thickness	1.5	mm
	$R_{\mathrm{AV}}$, $R_{\mathrm{MV}}$	Valve resistances	$10^{4}$	kg/(m·s)
	$\Delta t_{\mathrm{MV}}^{\text{open}}$	MV opening ramp duration	10	ms
	$\Delta t_{\mathrm{MV}}^{\text{close}}$	MV closing ramp duration	35	ms
	$\Delta t_{\mathrm{AV}}^{\text{open}}$	AV opening ramp duration	10	ms
	$\Delta t_{\mathrm{AV}}^{\text{close}}$	AV closing ramp duration	80	ms
Domain displacement	$\mu_{\mathrm{ALE}}$	Fluid domain stiffness	0.01	Pa
	$\kappa_{\mathrm{ALE}}$	Fluid domain bulk modulus	0.01	Pa

**TABLE A4:** Left heart test case. Parameters for the solid mechanics model. For the meaning of the parameters, we refer to Reference [42].

Subdomain	Parameter	Description	Value	Unit
	$\rho_{s}$	Solid density	$10^{3}$	kg/m^3^
	$b_{f}$	Guccione fiber parameter	8	—
	$b_{s}$	Guccione sheet parameter	6	—
	$b_{n}$	Guccione normal parameter	3	—
	$b_{\mathrm{fs}}$	Guccione fiber‐sheet parameter	12	—
	$b_{\mathrm{fn}}$	Guccione fiber‐normal parameter	3	—
	$b_{\mathrm{sn}}$	Guccione sheet‐normal parameter	3	—
	$\kappa_{G}$	Guccione bulk modulus	$5\cdot 10^{4}$	Pa
Left ventricle	$n_{f}$	Contraction ratio along fibers	1	—
	$n_{n}$	Contraction ratio along normal	0.4	—
	c	Stiffness	$0.88\cdot 10^{3}$	Pa
	$p_{\mathrm{LV}}^{0}$	End‐diastolic left ventricle pressure	8.63	mmHg
	$p_{\mathrm{LV}}^{\mathrm{ref}}$	Reference left ventricle pressure	7.5	mmHg
Left atrium	$n_{f}$	Contraction ratio along fibers	1	—
	$n_{n}$	Contraction ratio along normal	0.4	—
	c	Guccione stiffness	$1.76\times 10^{3}$	Pa
	$p_{\mathrm{LA}}^{0}$	End‐diastolic left atrium pressure	9	mmHg
	$p_{\mathrm{LA}}^{\mathrm{ref}}$	Reference left atrium pressure	6.75	mmHg
Aorta	$\kappa_{\mathrm{NH}}$	neo‐Hooke bulk modulus	$10\cdot 10^{5}$	Pa
	$\mu_{\mathrm{NH}}$	neo‐Hooke stiffness	$5.25\cdot 10^{5}$	Pa
	$p_{\mathrm{Ao}}^{0}$	End‐diastolic aortic pressure	71.26	mmHg
Atrioventricular ring	$\kappa_{\mathrm{NH}}$	neo‐Hooke bulk modulus	$50\cdot 10^{5}$	Pa
	$\mu_{\mathrm{NH}}$	neo‐Hooke stiffness	$10\cdot 10^{5}$	Pa
Boundary conditions	$K_{\mathrm{epi}}$	Pericardium normal stiffness	$2\cdot 10^{5}$	Pa/m
	$C_{\mathrm{epi}}$	Pericardium normal viscosity	$2\cdot 10^{3}$	Pa·s/m
	$K_{\mathrm{epi}}^{\mathrm{fat}}$	Epicardial fat normal stiffness	$2\cdot 10^{2}$	Pa/m

**TABLE A5:** Left heart test case. Parameters for the circulation model. We refer to [25] for the meaning of each parameter.

Compartment	Parameter	Description	Value	Unit
Right atrium	$E_{A}$	Active elastance	0.06	mmHg/mL
	$E_{P}$	Passive elastance	0.07	mmHg/mL
	$t_{C}$	Relative duration of contraction	0.17	—
	$t_{R}$	Relative duration of relaxation	0.17	—
	$V_{\text{rest}}$	Resting volume	4	mL
	$V_{0}$	Initial volume	64.1702	mL
Right ventricle	$E_{A}$	Active elastance	0.55	mmHg/mL
	$E_{P}$	Passive elastance	0.05	mmHg/mL
	$t_{C}$	Relative duration of contraction	0.34	—
	$t_{R}$	Relative duration of relaxation	0.15	—
	$V_{\text{rest}}$	Resting volume	16	mL
	$V_{0}$	Initial volume	148.9384	mL
Pulmonary arteries	R	Resistance	0.05	mmHg·s/mL
	C	Capacitance	10.0	mL/mmHg
	$R_{\mathrm{up}}$	Upstream resistance	0.0	mmHg·s/mL
	L	Inductance	$5\cdot 10^{-4}$	mmHg·s/mL^2^
	$p_{0}$	Initial pressure	20	mmHg
	$Q_{0}$	Initial flow rate	69.32	mmHg
Pulmonary veins	R	Resistance	0.025	mmHg·s/mL
	C	Capacitance	38.4	mL/mmHg
	L	Inductance	$2.083\cdot 10^{-4}$	mmHg·s/mL^2^
	$p_{0}$	Initial pressure	17	mmHg
	$Q_{0}$	Initial flow rate	105.52	mmHg
Systemic arteries	R	Resistance	0.45	mmHg·s/mL
	C	Capacitance	2.19	mL/mmHg
	$R_{\mathrm{up}}$	Upstream resistance	0.07	mmHg·s/mL
	L	Inductance	$2.7\cdot 10^{-3}$	mmHg·s/mL^2^
	$p_{0}$	Initial pressure	80	mmHg
	$Q_{0}$	Initial flow rate	66.58	mmHg
Systemic veins	R	Resistance	0.26	mmHg·s/mL
	C	Capacitance	60.0	mL/mmHg
	L	Inductance	$5\cdot 10^{-4}$	mmHg·s/mL^2^
	$p_{0}$	Initial pressure	30.90	mmHg
	$Q_{0}$	Initial flow rate	89.63	mmHg
